# The Impact of Vulvar Cancer on Psychosocial and Sexual Functioning: A Literature Review

**DOI:** 10.3390/cancers14010063

**Published:** 2021-12-23

**Authors:** Francesca Malandrone, Federica Bevilacqua, Mariagrazia Merola, Niccolò Gallio, Luca Ostacoli, Sara Carletto, Chiara Benedetto

**Affiliations:** 1 Department of Clinical and Biological Sciences, University of Torino, 10043 Torino, Italy; francesca.malandrone@unito.it (F.M.); luca.ostacoli@unito.it (L.O.); 2 Gynaecology and Obstetrics 1, Department of Surgical Sciences, University of Torino, City of Health and Science, 10126 Torino, Italy; federica.bevilacqua@unito.it (F.B.); niccolo.gallio@unito.it (N.G.); chiara.benedetto@unito.it (C.B.); 3 Clinical Psychology Service, City of Health and Science, 10126 Torino, Italy; sara.carletto@unito.it; 4 Department of Neuroscience “Rita Levi Montalcini”, University of Torino, 10126 Torino, Italy

**Keywords:** vulvar cancer, anxiety, depression, distress, sexual functioning, quality of life

## Abstract

**Simple Summary:**

The diagnostic and therapeutic pathway of vulvar cancer impacts severely on the psychosocial and psychosexual equilibrium of women affected by it. The current literature shows the presence of depressive and anxious symptoms in association with physical, psychological and behavioural alterations in sexuality as well as deterioration of partner relationship. The aim of this article is to highlight the difficulties and challenges faced by women diagnosed and treated for vulvar cancer to provide early recognition and appropriate assistance. By implementing an integrated care model, it should be possible to detect unmet needs and improve the quality of life of these women.

**Abstract:**

Women who are diagnosed and treated for vulvar cancer are at higher risk of psychological distress, sexual dysfunction and dissatisfaction with partner relationships. The aim of this article is to provide a review of the psychological, relational and sexual issues experienced by women with vulvar cancer in order to highlight the importance of this issue and improve the quality of care offered to these patients. A review of the literature was performed using PubMed, CINAHL, PsycINFO, and the Cochrane Library. The results are presented as a narrative synthesis and highlight the massive impact of vulvar cancer: depressive and anxiety symptoms were more frequent in these women, and vulvar cancer may have a negative effect on sexuality from a physical, psychological and behavioural point of view. Factors that may negatively affect these women’s lives are shame, insecurity or difficulties in self-care and daily activities. This review highlights the psychosocial and psychosexual issues faced by women diagnosed and treated for vulvar cancer, although more studies are needed to better investigate this field of interest and to identify strategies to relieve their psychological distress. Care providers should implement an integrated care model to help women with vulvar cancer recognise and address their unmet needs.

## 1. Introduction

Vulvar cancer is a rare malignancy with an incidence of 2.5–4.4 per 100,000 persons per year, making it the fourth most common gynaecological malignancy in Europe [1]. The most common type is vulvar squamous cell carcinoma (VSCC), followed by basal cell carcinoma, extramammary Paget’s disease and vulvar melanoma. The median age at diagnosis is 69 years [2]. Risk factors for the development of VSCC include increasing age, human papilloma virus (HPV) infection, immunodeficiency, smoking and vulvar inflammatory conditions [3]. Notably, in recent decades, the incidence of vulvar intraepithelial neoplasia (VIN), the precursor to VSCC, has doubled for all age groups, increasing the most for patients under the age of 50 [4].

VSCC has two precursor forms: (1) vulvar intraepithelial neoplasia (VIN) HPV-related (i.e. vulvar high-grade squamous intraepithelial lesion, VHSIL) and (2) HPV-unrelated, also known as differentiated VIN (dVIN), typically related to chronic vulvar inflammatory conditions (e.g., lichen sclerosus or lichen planus). These two different biological entities also have a differing epidemiology, characteristics, and prognosis [5,6,7,8]. The diagnosis of vulvar cancer is often delayed, as there is not adequate awareness among women: the majority of women feel embarrassed to ask their physicians about vulvar health and vulvar symptoms [9]. VSCC is often asymptomatic for a long period of time, or it can present with pruritus, irritation, or pain. Late-presenting symptoms include bleeding, pain, vaginal discharge, and urinary- or bowel-related symptoms. Clinically, VSCC can present as an erythematous patch, plaque, ulcer, or mass.

Staging is based on a vulvar biopsy to determine stromal invasion, which represents an important prognostic factor, then a clinical assessment of tumour size, groin lymph nodes and eventual distant metastases [10]. The treatment of stage I disease is surgical excision with adjuvant radiation (RT) in cases with high-risk factors. Stages II–IVA vulvar cancer, locally advanced disease, is usually treated with radical surgery and adjuvant chemoradiation. In selected cases, neoadjuvant chemoradiation can be used to reduce tumour size to facilitate surgical resection in an attempt to avoid a pelvic exenteration. For stage IVB, palliation with chemotherapy (CT) and/or RT is recommended [10].

The various surgical options have different impacts on the quality of life of the patients. A wide local excision is a simple excision of a vulvar tumour, a procedure reserved for preinvasive disease and stage IA vulvar cancers; this is the most conservative vulvar surgery in cases of malignancy. A modified radical vulvectomy combines the excision of the primary tumour and bilateral groin dissection [11]. On incision at the primary vulvar tumour site, the surgeon should try to spare the vital organs (e.g., urethra, clitoris and anal sphincter) [12]. Reconstructive surgery may be needed during this procedure [13]. Wound dehiscence and infection are common after radical vulvectomy. The most radical surgery is total pelvic exenteration, which is reserved exclusively for carefully selected patients with malignancy extended to other organs (e.g., urethra, anus and vagina). Despite its large impact on the patient’s quality of life, pelvic exenteration can be a potentially curative option. Surgical morbidity is high and includes infections, wound dehiscence, and urinary- and gastrointestinal-related complications. Inguinofemoral lymph node dissection is indicated when stromal invasion is >1 mm, and it is burdened with many postoperative complications including a significantly increased risk of lymphedema and wound breakdown [14]. For this reason, sentinel lymph node biopsy should be considered in pT1 vulvar cancers [15].

Although vulvar surgery and treatment has become more targeted and less radical over the decades, it may still cause scarring and mutilation of the external genitalia, and it also may affect various nerves and blood vessels involved in sexual, anal and/or urinary functions [16]. Women who undergo surgical treatment for vulvar cancer or VIN are at high risk of psychological distress, sexual dysfunction and dissatisfaction with partner relationships [17]. Factors associated with post-treatment sexual dysfunction include history of depression or anxiety, patient’s increased age and the excision size of the vulvar cancer. Interest in the QoL of women with vulvar cancer has increased in recent years [18,19,20]. Most studies on QoL after vulvectomy are, however, focused on postoperative complications and long-term side effects [21,22,23], whereas the impact that surgery may have on sexual health and on a patient’s relationships have not been properly investigated. Despite the first paper on post-surgical sexual function following vulvar cancer being published almost 40 years ago [24], the true impact of the different types of vulvectomies on the sexual health of vulvar cancer survivors has been poorly investigated.

The aim of this literature review is to provide a comprehensive synthesis of the psychological, relational and sexual issues experienced by women with vulvar cancer in order to inform both researchers and clinicians of the steps needed to advance knowledge in this area and to improve the quality of care offered to these patients.

## 2. Material and Methods

The search was conducted in the following databases on 14 July 2021: PubMed, CINAHL, PsycINFO and the Cochrane Library. Keywords included: vulvar malignancy, vulvar neoplasms, vulvar cancer, vulva *, vulvectomy, gynaecologic *, gynecologic *, cancer *, tumor *, cancer survivors/psychology, psychological adaptation, quality of life, psychological distress, stress, anxiety, depression, sexuality, sexual dysfunction, partner and sexual partner. Primary research studies that investigated psychological, psychosocial and/or sexual consequences of vulvar cancer in adult women were included with no limitations related to publication date. Studies on gynaecological cancer not focusing specifically on vulvar malignancy were excluded. Only studies in the English language were included. The references of the identified studies and relevant reviews were manually searched to identify other relevant articles. The results of the evidence found are presented as a narrative synthesis.

## 3. Results

**Figure 1:**
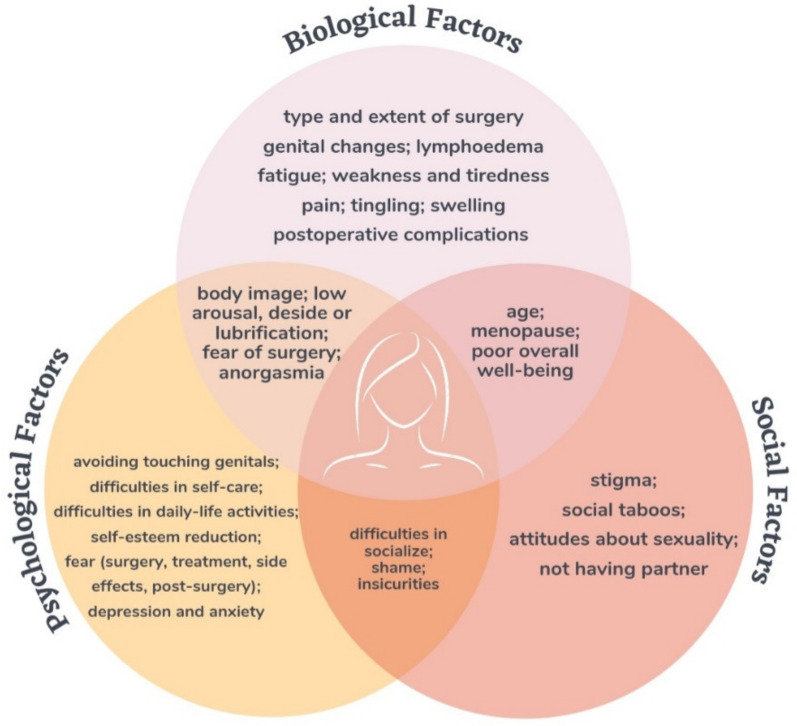
Summary of the results. This figure shows the main bio–psycho–social factors that impact the psychosocial and sexual well-being of women with vulvar cancer.

The initial search retrieved 1715 articles after duplicates removal. Of these, only 30 articles were considered eligible for inclusion in the review. The details of the included studies are described in Table 1, while a summary of the results is given in Figure 1.

**Table 1:** Overview of the included studies.

Category	Studies	Country	Mean Age	Type of Surgery	Measures	Results
Quality of life	Blbulyan et al., 2020 [25]	Russia	56.3	-	EORTC; FACT-G	Lower overall quality of life. Restrictions in physical activity, poorer social interaction and emotional sphere. Worse global health status.
	de Melo Ferreira et al., 2012 [26]	Brazil	66.9	Vulvectomy + IFL	EORTC	
	Farrel et al., 2014 [27]	Australia	63	IFL	UBQC	
	Gane et al., 2018 [28]	Australia	57	Vulvectomy with or without SNB or IFL	FACT-G	
	Günther et al., 2014 [29]	Germany	63 WLE–59 RV	WLE or radical vulvectomy with or without IFL	EORTC	
	Hellinga et al., 2018 [30]	Netherlands	65.5	WLE/radical vulvectomy/pelvic exenteration + reconstruction with lotus petal flap	EORTC	
	Janda et al., 2004 [18]	Australia	68.8	WLE or radical vulvectomy with or without IFL	ECOG-PSR; FACT-G	
	Jones et al., 2016 [31]	UK	59.9	WLE or radical vulvectomy with or without IFL	EORTC	
	Likes et al., 2007 [32]	USA	47.5	WLE	EORTC	
	Oonk et al., 2009 [20]	Netherlands	69	WLE or radical vulvectomy with SNB or IFL	EORTC	
	Novackova et al., 2012 [33]	Czech Republic	66.5 CONS–73.8 RAD	WLE or radical vulvectomy with SNB or IFL	EORTC	
	Senn et al., 2013 [34]	Germany	18 (VIN) 42 (K)	Laser vaporisation/WLE/vulvectomy/radical vulvectomy/exenteration with or without SNB or IFL	WOMAN-PRO	
	Weijmar Schultz et al., 1990 [35]	Netherlands	55	WLE or radical vulvectomy with or without IFL	ad hoc questionnaire	
	Trott et al., 2020 [36]	Germany	63	Unspecified vulvar surgery with or without SNB or IFL with or without reconstruction	EORTC	
Partner relationship	Aerts et al., 2014 [37]	Belgium	57.4	Vulvectomy with or without SNB	DAS	Lower quality of partner relationship, marital satisfaction and dyadic cohesion.
	Barlow et al., 2014 [38]	Australia	58	Radical partial or total vulvectomy with or without IFL	clinical interview	
Sexual Functioning	Aerts et al., 2014 [37]	Belgium	57.4	Vulvectomy with or without SNB	SFSS; SSPQ	Worse sexual functioning. Disruption and reduction in sexual activity.
	Andersen et al., 1983 [24]	USA	55	WLE or radical vulvectomy	SCL-90	
	Andersen et al., 1988 [39]	USA	50.3	Laser vaporisation/WLE/vulvectomy	DSFI; SAI	
	Andreasson et al., 1986 [40]	Denmark	45.8	Vulvectomy	ad hoc questionnaire	
	Barlow et al., 2014 [38]	Australia	58	Radical partial or total vulvectomy with or without IFL	clinical interview	
	Blbulyan et al., 2020 [25]	Russia	56.3	-	FSFI	
	Farrel et al., 2014 [27]	Australia	63	IFL	clinical information	
	Green et al., 2000 [41]	USA	60	Vulvectomy with or without IFL	ad hoc questionnaire	
	Grimm et al., 2016 [42]	Germany	51.5	Laser vaporisation/WLE/radical vulvectomy	FSFI	
	Hazewinkel et al., 2012 [43]	Netherlands	68	WLE or radical vulvectomy with or without SNB or IFL	FSFI; BIS	
	Hellinga et al., 2018 [30]	Netherlands	65.5	WLE/radical vulvectomy/pelvic exenteration + reconstruction with lotus petal flap	FSFI; BIS	
	Jones et al., 2016 [31]	UK	59.9	WLE or radical vulvectomy with or without IFL	clinical interview	
	Weijmar Schultz et al., 1990 [35]	Netherlands	55	WLE or radical vulvectomy with or without IFL	ad hoc questionnaire	
	Likes et al., 2007 [32]	USA	47.5	WLE	FSFI	
Psychological health	Aerts et al., 2014 [37]	Belgium	57.4	Vulvectomy with or without sentinel node dissection	BDI	Presence of depressive and anxious symptoms, worsened by altered body image and sexual difficulties. Impact on general well-being, quality of life, and relationship with partner and families.
	Avery et al., 1974 [44]	USA	NA	Vulvectomy	clinical information	
	Andersen et al., 1983 [24]	USA	55	WLE or radical vulvectomy	BDI	
	Andreasson et al., 1986 [40]	Denmark	45.8	Vulvectomy	ad hoc questionnaire	
	Corney et al., 1992 [45]	UK	71% >65	Radical vulvectomy, Wertheim’s hysterectomy or pelvic exenteration	HADS; clinical interview	
	Green et al., 2000 [41]	USA	60	Vulvectomy with or without IFL	PRIME-MD	
	Janda et al., 2004 [18]	Australia	68.8	WLE or radical vulvectomy with or without IFL	FACT-G; HADS	
	Jefferies and Clifford, 2012 [46]	UK	>50	-	clinical interview	
	McGrath et al., 2013 [47]	Australia	NA	-	clinical interview	
	Senn et al., 2011 [48]	Germany	55	Laser vaporisation/WLE/radical vulvectomy with or without SNB or IFL	clinical interview	
	Senn et al., 2013 [34]	Germany	18 (VIN) 42 (K)	Laser vaporisation/WLE/vulvectomy/radical vulvectomy/exenteration with or without SNB or IFL	WOMAN-PRO	
	Stellman et al., 1984 [49]	USA	53.4	Vulvectomy or radical vulvectomy	SQ	
	Tamburini et al., 1986 [19]	Italy	51.7	Vulvectomy with or without IFL	clinical interview	
	Thuesen et al., 1992 [50]	Denmark	41.4	WLE	clinical interview; ad hoc questionnaire	

BDI = Beck Depression Inventory; BIS = Body Image Scale; ECOG-PSR = ECOG Scale of Performance Status; CONS = sentinel lymph node biopsy; DAS = Dyadic Adjustment Scale; DSFI = Derogates Sexual Functioning Inventory; EORTC = European Organization for Research and Treatment of Cancer; FACT-G = Functional Assessment of Cancer Therapy—General; FSFI = Female Sexuality Index; GSI = Global Severity Index; HADS = Hospital Anxiety and Depression Scale; IFL = inguinofemoral lymphadenectomy; NA = not available; PRIME-MD = Primary Care Evaluation of Mental Disorders; RAD = inguinofemoral lymphadenectomy; RV = radial vulvectomy; SAI = Sexual Arousability Index; SCL-90 = Symptoms Checklist-90; SFSS = Short Sexual Functioning Scale; SNB = sentinel node biopsy; SSPQ = Specific Sexual Problems Questionnaire; SQ = Kellner Symptom Questionnaire; UBQC = Utility-Based Questionnaire-Cancer; WLE = wide local excision.

### 3.1. Psychological Impact 

Vulvar cancer is a rare condition. Due to the lack of studies regarding the impact of the disease, little is known about the specific emotional, social and psychological impacts on these patients. Diagnosis and treatment, primarily consisting of surgery ranging from local excision to radical vulvectomy and clitoris removal, may have a significant negative psychological effect on these women. Symptoms may range from anxiety and sexual dysfunction to major depressive disorders. Depending on the extent of surgery, participants’ self-perception of being a woman has been reported to be influenced in at least four dimensions: the appearance of post-surgical female genitals, sexuality, attractiveness and self-confidence [48].

The need for emotional support from the preoperative phase to follow-up care has already been recognised by Avery et al. in 1974 [44]. The first systematic review was carried out by Jefferies and Clifford [51], who examined the psychological, physical and sexual consequences for women following diagnosis and treatment for cancer of the vulva. Eight out of the 14 studies analysed reported psychological changes as a result of the diagnosis and surgery for vulvar cancer [19,24,39,40,41,45,49,50]. Only a few of these authors used validated measurement tools to record levels of depression and anxiety [18,24,37,41,45,49].

In terms of psychological distress and/or depression, in the study by Andersen [24], women affected by vulvar cancer experienced substantial and significant levels of distress in comparison to healthy women. Nearly 50% of the patients interviewed by Andreasson et al. [40] had an altered sense of their body image, describing feelings of “not being the same woman”. This finding was later supported by Andreasson et al. [40], Stellman et al. [49], and Thuesen et al. [50].

Stellman and colleagues [49] reported that four out of nine women were unable to name the anatomic area surgically removed. This may have increased the women’s feelings of isolation and embarrassment. The study also reported that six out of nine women were depressed and anxious. Loss of self-confidence and self-esteem, also associated with depression, were noted.

The study by Tamburini et al. [19] revealed that 72% of the sample showed symptoms on the hypochondria scale, 63% on the depression scale, 63% on the hysteria scale and 18% on the psychotic scale (paranoia, schizophrenia). These results, both for “neurotic” (hypochondria, hysteria and depression) and, to a lesser extent, the “psychotic” scales (paranoia, schizophrenia) tended to be more pathological than in patients undergoing the same radical surgery for carcinoma of the uterine cervix. This may be explained by the feeling of awkwardness or loss of self-esteem resulting from external genital mutilation and by severe sexual difficulties.

In a retrospective study by Corney et al. [45], women who underwent major gynaecological surgery (vulvectomy, hysterectomy and pelvic exenteration) for carcinoma of the cervix and vulva were interviewed to evaluate postoperative psychosocial and psychosexual problems, revealing that 21% of the women suffered from anxiety and 14% from depression.The study also tried to assess the relationship between the level of distress and the different clinical phases, finding that the period of highest distress or worry usually coincided with the period of most uncertainty. For 39% of women, the most distressing time was between the first medical indication of clinical problems and the diagnosis of cancer, and an additional 37% felt that it was the period between diagnosis and the operation. Moreover, this study showed that the presence of sexual problems was significantly associated with the women’s level of anxiety. However, it was difficult to ascertain whether the sexual problems were making the women more anxious or whether their anxiety was affecting their sexual behaviour, cognition or emotions.

Green [41] detected symptoms of depression in 31% of women treated with vulvar surgery, but only 14% were taking antidepressant medication. Women with higher depression scores had greater sexual aversion disorder and experienced higher levels of body image disturbance and global sexual dysfunction. Janda et al. [52] showed that 21.8% of patients in the sample were affected by severe anxiety and 6.3% by depression.

The study by Senn et al. [48] focused on the symptoms of women during the first 6 months following surgical treatment for vulvar neoplasia. Using narrative interviews, the study showed eight interrelated psychological themes: delayed diagnosis, disclosed disease, disturbed self-image, changed vulva care, experienced wound-related symptoms, evoked emotions, affected interpersonal interactions and feared illness progression. The unknown diagnosis, surgery, location, changed female genitals and experienced symptoms evoked feelings of embarrassment, uncertainty, fear, sadness and tiredness. Senn et al. [34] tried to measure these symptoms with the WOMAN-PRO instrument, developed by them in 2013. The results showed that the three most prevalent psychosocial symptoms/issues were “tiredness” (95.4%), “insecurity” (83.1%) and “feeling that my body has changed” (76.9%). Despite physical symptoms occurring more frequently, in this sample they were less distressing than difficulties in daily life and psychosocial symptoms/issues.

Through a phenomenological study, Jefferies and Clifford [46] gave a voice to women regarding the stories of their illness, their feelings and their thoughts about diagnosis and treatment for cancer of the vulva. This study was an overview of their lived experience described using the concept of invisibility in the context of four existential dimensions: body, relationship, space and time.

The findings of the study by McGrath et al. [47] demonstrated that the challenges in relation to the diagnosis and treatment of vulvar cancer are exacerbated by concerns about privacy, shame and fear. Because of the private nature of the disease, the women from the sample tended to keep the condition a secret once diagnosed; the feeling of shame was as powerful as the sense of privacy. These feelings were so strong that even when a woman died of this cancer, the type of cancer was not revealed to avoid shame. Lastly, it was common for the women to feel scared and worried about the cancer, especially when first diagnosed.

In the study by Aerts et al. [37], women with a diagnosis of vulvar malignancy were compared to healthy controls. When compared with the situation before surgery, no significant differences in depressive symptoms, general well-being and quality of partner relationship were found after surgery. However, in comparison with healthy controls, women with vulvar malignancy reported significantly lower levels of psychological functioning both before and after treatment.

Finally, the review of the literature by Boden et al. [53] highlighted important psychosocial issues that women diagnosed and living with cancer of the vulva have to face. Challenges include social stigma surrounding the vulva and the diagnosis of vulvar cancer, feeling unprepared both physically and psychologically and a lack of information and support.

The literature, although poor, shows the massive impact of vulvar cancer, both in psychological and social dimensions. Through different means of investigation, the presence of depressive and anxiety symptoms in women has been demonstrated in every phase, from diagnosis to postoperative follow up. Moreover, the altered body image due to the surgical treatment and the presence of sexual difficulties can lead to deterioration in the emotional state. All of these elements can influence the women’s general well-being, quality of life and their relationship with partners and families.

In conclusion, the review of the literature reveals the paucity of current studies regarding women who are suffering from cancer of the vulva today. Clearly there is a need for more research into the special needs of this small group of women.

### 3.2. Quality of Life, Sexuality and Partner Relationship

Several studies [18,27,28,31,32,33,36] reported an overall worsening of quality of life. Three studies [25,29,30] explored this aspect further, noting that the worsening of quality of life was related to a decrease in physical and cognitive functioning, social interactions and an increase in physical and emotional symptoms. The study by de Melo Ferreira and colleagues [26], going into more detail, found a negative correlation between quality of life and the severity of lymphoedema of the lower extremities.

Only two studies investigated partner relationships [37,38]. Aerts and colleagues [37] observed that poorer quality of partner relationship, marital satisfaction and dyadic cohesion were more common in women with preoperative vulvar malignancy. Dissatisfaction with partner life was also maintained at 6 months and 1 year after the operation. In this respect, Barlow and colleagues [38] found that conservative surgery led to no negative impact on couples’ relationships.

Sexual functioning in this disease has been investigated but most frequently from a physiological perspective. However, some studies have intersected biological function with more psychosocial components. Aerts and colleagues [37] found that there is a correlation between psychosocial well-being and sexual dysfunction in patients with vulvar cancer. In general, it seems that vulvar cancer has a negative effect on sexuality not only from a physical point of view, for example, due to the fact of presenting with anorgasmia, difficulty in lubrication or pain, but also from a psychological and behavioural point of view, e.g., due to the fact of reporting reduced desire, reduced satisfaction, reduced sexual activity, fear of penetration or avoidance behaviour [24,25,27,30,31,32,35,38,40,41,43]. Of interest are the results presented in the study by Andersen and colleagues [24], which showed that while there was a deterioration in sexual functioning from a physiological point of view, there was little negative impact on sexual life. However, there was a reluctance to enter into relationships with new partners for those who did not have any before the illness.

#### Related Bio–Psycho–Social Factors

Several factors are responsible for the deterioration of quality of life, sexual functioning and partner relationships. In general, studies have considered the various factors, often incorporating the collection of these data into clinical interviews or using generic, albeit validated, assessment instruments. Although pre-existing problems have been shown to play an important role [32,33,36], several studies have reported that treatments and surgeries are crucial to patients’ physical, mental and social health. Three studies [24,41,42] pointed out that outcomes seem to be correlated with the magnitude of surgical intervention. Aerts and colleagues and Likes and colleagues [32,37] found a negative correlation between quality of life and the excision size of the vulvar malignancy, meanwhile Gunther et al. [29], Barlow et al. [38] and Hazewinkel et al. [43] identified radical vulvectomy and clitoral removal as determinants of worsening quality of life, sexual functioning and partner life. Inguinofemoral lymphadenectomy also appears to negatively impact the lives of vulvar cancer patients as was found by Novackova and colleagues [33].

Some authors have observed that in connection with surgery, important impacting factors are fear of possible removal of their clitoris and fear of pain on resumption of sexual intercourse [38]. In general, it seems that aesthetic and functional changes of the genitals [39] and having undergone multiple vulvar procedures [38] have a significantly negative impact on well-being during the postoperative period. The postoperative period itself is not without risk due to the possibility of incurring postoperative wound-healing complications [36] that not only slow down the healing process but also hinder the recovery of normal biological, social and psychological functions. Factors associated with post-treatment sexual dysfunction include older age, poor overall well-being and history of depression and anxiety [37,42].

Regarding therapies, two studies found that radiotherapy or adjuvant inguinal radiotherapy [33,43] have a negative impact on patients’ lives, mainly due to the associated side effects. The most impactful side effect seems to be lymphoedema, considered globally [20,29,31,33,38] or specifically of the lower extremities [18,26]. Lymphoedema causes patients pain, changes in sleep, fatigue, reduced movement amplitude and financial costs [26]. It is a tiring and energy-reducing condition, as it requires constant attention in terms of wearing compression stockings, performing massages and undergoing other treatments. It can reduce a patient’s ability to work, perform household duties and socialise as well as negatively affecting the patients’ body image and self-esteem [18]. Other side effects that affect patients’ lives are pain [28,29,30,31] and leg pain [27], persistent swelling of the lower limbs or vulva and/or pelvic/abdominal region [28], tingling [28] and fatigue [31,33].

From a psychological and behavioural point of view, factors such as body image disruption [24,34,38,41], feelings of weakness and fatigue [28,31,34] and avoidance behaviours regarding sexual intercourse and touching the genital area [38] seem to be decisive in worsening quality of life, quality of relationships and sexuality.

In addition to the psychological aspects of vulvar cancer, social, cultural and interpersonal aspects negatively affect patients’ quality of life, sexuality and relationship with their partners. Studies have shown that age can modulate a person’s response to the disease and treatment. On the one hand, younger women show more distress because of the greater proximity to the onset of menopause [24] and the impediments in the activities of daily living: the younger the patients are, the more they feel they should and would like to have more active lives with a greater number of relationships and a more active sex life [34]. On the other hand, it has been observed that with advancing age, patients experience greater suffering related to difficulties in investing in new romantic relationships and taking care of themselves [32,33,36]. Older patients also have prevalent attitudes about sexuality in later life such as those indicating that such activity is inappropriate, unimportant or readily expendable [24]. More generally, factors that negatively impact the lives of women with vulvar cancer are shame or insecurity [33,34], difficulties in self-care [48] and in daily activities such as homecare [34] and not having a sexual partner [33]. Stigma and social taboos also seem to play a key role [48].

## 4. Discussion

Despite covering a large time span—from the 1983 study by Andersen and colleagues to the 2020 study by Blbulyan et al.—the impact of vulvar cancer on mental health, quality of life, sexuality and relationships has been little investigated. The aim of this narrative review was to update the knowledge on these themes and suggest future directions for clinical practice and research.

Regarding quality of life, studies have often focused on well-being in general or global health; however, specific aspects of quality of life, emotional regulation and the ability and willingness to take care of oneself would also be of interest. Vulvar cancer seems to also have a role in worsening sexual functioning with a disruption and reduction in sexual activity. Similarly, the included studies observed lower quality of partner relationships, marital satisfaction and dyadic cohesion.

Regarding mental health issues, depressive and anxiety symptoms were prevalent among women affected by vulvar neoplasia at every step of the diagnostic and therapeutic pathway. Different factors can contribute to the onset of these symptoms. Psychological distress may be related to the altered body image due to the aftermath of the surgery. The results of the studies, although scarce, demonstrate that women may experience feelings of shame and embarrassment related to strong social stigma, which may lead to feelings of isolation and a loss of self-esteem. Moreover, sexual dysfunction is strongly associated with anxiety and depressive symptoms, which are often clinically significant, with a mutual influence.

Depression and cancer risk seem to be linked by a mutual relationship. The experience of receiving a cancer diagnosis can be a significant source of distress, with the onset of anxiety and/or depressive symptoms that can lead to sleep disturbance which may, in turn, increase the risk of depression. Major depressive disorder (MDD) is common among cancer patients with prevalence rates up to four-times higher than the general population [54]. Conversely, depression confers worse outcomes in oncological settings including non-adherence to treatment and increased mortality [55]. According to a study by Wang et al. [56], the estimated absolute risk increases (ARIs) associated with depression and anxiety are 34.3 events/100,000 person years for cancer incidence and 28.2 events/100,000 person years for cancer-specific mortality. Several mechanisms could explain this reciprocal influence [56]. Psychosocial stressors in cancer promote inflammation and oxidative/nitrosative stress, with alterations in cytokine secretion and regulation (TNF-a or Il-6) [57]; decreased immunosurveillance; dysfunctional activation of the autonomic nervous system and hypothalamic–pituitary–adrenal axis. Given the high prevalence of depression and anxiety in the general population, particularly among cancer patients, and in consideration of the bidirectional link between the neuroendocrine and immune systems, the screening and intervention of underlying depressive and anxiety symptoms has significant repercussions both on clinical practice and public health regarding cancer prevention and treatment.

The studies included in this review have several limitations. First, most of the studies use non-validated tools such as clinical interviews or data taken from medical records. While these have made it possible to obtain qualitative insight into patients’ experiences, there is less scope for distinguishing between areas and isolating the factors that impact on them. Sexual function has often been addressed on a physiological level via the maintenance of sexual activity or physical dysfunction (e.g., orgasm, pain on penetration and lubrication). Sexual satisfaction cannot be reduced simply to the extent of physical impairment, the presence of symptoms or the ability to perform a sexual task considered normal [35]. The fact that sex life is scarcely investigated from a psychological point of view may be due not only to the limitations of the studies but also to social and cultural issues surrounding the perception of the possibility for women to desire an active and satisfying sex life after the age of 50. A healthy sex life has been shown to play a key role in maintaining mental and physical health.

### 4.1. Implications for Future Research

Future studies with better methodological quality are needed to obtain more reliable data. In particular, controlled studies are needed. Given the high survival rate of vulvar cancer patients [58], quality of life, sexuality and psychological well-being are key areas for investigation, along with the long-term consequences of the disease and treatment pathways. These areas are crucial not only due to the high prevalence of psychological distress among cancer patient but also due to the consequences of such disorders on overall health [59]. In fact, considering breast cancer, a much more widespread, well-known and well-studied type of cancer among women, a large volume of literature is available on quality of life [60], sexuality [61,62] and partner relationships [63,64]. Furthermore, future studies should investigate the psychological aspects of sexual experience, shame, body image, desire and avoidance of desire as well as considering the sexual orientation of patients and the specific needs related to it that may emerge. It would also be useful to consider sexuality as a subjective, personal and individual dimension and not only within the context of the couple. Regarding the relationship with a partner, it would be interesting in the future to investigate not only the level of satisfaction and sexual activity but also other factors such as attachment.

With regard to the impact of different medical treatments, the approach to vulvar cancer has changed considerably over time, with a succession of different surgical procedures and pharmacological treatments. In light of this, it would be interesting to assess how different types of intervention may have different impacts on psychological, social and sexual outcomes. Future studies could compare different treatment pathways to explore this further.

### 4.2. Implications for Clinical Practice

The impacts of vulvar cancer on psychosocial- and sexuality-related areas highlight the importance of implementing effective strategies in both primary and secondary prevention. The ideal goal should be early recognition and appropriate treatment of the elements of psychosexual and psychosocial distress. These approaches should lead to an improvement in general well-being, a healthier sex life and more stable and supportive relationships.

Firstly, healthcare professionals should be adequately trained to help women to understand the basics of female external genital anatomy, so that they can learn the difference between physiological and pathological features. Very few women engage in vulvar self-examination, and few women who identify abnormalities seek appropriate medical care. Vulvar self-examination may allow women to have a healthier relationship with their genitals, overcoming the obstacles due to the feelings of shame, judgment and embarrassment. In fact, this is an easy procedure that takes only a few minutes and could change the clinical course of some pathologies and, in some cases, could save lives [9].

The National Comprehensive Cancer Network has recognised psychological distress as the sixth vital sign in cancer care [65]. For this reason, it may be useful to facilitate its early detection using screening tests in a very early phase of the diagnostic and therapeutic pathways. This kind of test should be approved and validated so that it can determine clinical risk categories and assign the most effective interventional treatment. The importance of the role of psycho-oncologists in the treatment of vulvar cancer should be emphasised for proposing and offering psychological support and psychoeducational interventions. These kinds of interventions could be useful not only for sustaining women’s sexual health [66] and their emotional condition but also for supporting the familial network. Moreover, evaluation by a psychiatric specialist may be useful in order to provide possible psychopharmacological therapy in the presence of severe depressive–anxiety symptoms, analysing the risk factors and any pharmacological interactions with chemotherapy treatments. This may be useful in improving the detection and treatment of psychosexual and psychosocial distress. The ideal goal could be the integration of a psycho-oncologist and/or psychiatrist in the multidisciplinary team for the treatment of vulvar cancer with the aim of addressing both the physical and psychosocial needs of these women [67]. 

## 5. Conclusions

This review highlights the psychosocial and psychosexual issues faced by women diagnosed with and treated for vulvar cancer. Many questions regarding the detection and management of psychological distress, sexual dysfunction and relational problems remain open, mainly due to the limited research into this area and the scarce integration of psycho-oncological knowledge in routine care. Care providers should implement an integrated care model to help women with vulvar cancer to recognise and address their still unmet needs, working within a bio–psycho–social framework.
